# ZNF473 as a biomarker and potential therapeutic target in cancer: integrated bioinformatics and experimental evidence with a focus on hepatocellular carcinoma

**DOI:** 10.3389/fonc.2026.1810579

**Published:** 2026-05-15

**Authors:** Yifan Ren, Tongxiang Wang, Yuqing Sun, Rui Zhang, Yan Wang, Jun Xu

**Affiliations:** Department of Hepatobiliary Surgery, First Hospital of Shanxi Medical University, Taiyuan, Shanxi, China

**Keywords:** cancer progression, HCC, immune, p-AKT, ZNF473

## Abstract

**Objective:**

Zinc finger protein 473 (ZNF473) has been implicated as a regulatory factor in several cancer types; however, its precise biological functions and underlying mechanisms remain incompletely defined. This study aimed to evaluate the biological and clinical relevance of ZNF473 in cancer through integrated bioinformatics analyses in combination with cell-based experimental validation.

**Methods:**

ZNF473 function was investigated using comprehensive bioinformatics approaches in combination with *in vitro* and *in vivo* experimental models.

**Results:**

ZNF473 exhibited differential expression across a range of normal tissues and malignancies and was significantly upregulated in more than 10 cancer types. Analyses based on the Tumor–Immune System Interaction Database indicated that ZNF473 expression correlated with both molecular and immune subtypes in multiple cancers. Receiver operating characteristic analyses yielded an area under the curve exceeding 0.7 in 19 cancer types, suggesting moderate diagnostic performance. Kaplan–Meier and Cox regression analyses identified significant prognostic associations in four cancer types, with hepatocellular carcinoma (HCC) demonstrating the most consistent clinical relevance in this dataset. In HCC, elevated ZNF473 expression was associated with unfavorable clinicopathological features, altered immune cell infiltration profiles, and enrichment of PI3K/AKT-related signaling pathways. Functional assays further showed that ZNF473 knockdown reduced cellular proliferation and migration, together with decreased levels of phosphorylated AKT (p-AKT), in liver tumor models.

**Conclusion:**

These findings support the role of ZNF473 as a clinically relevant biomarker candidate across cancers and suggest its involvement in HCC progression, potentially through activation of the AKT signaling pathway. The HCC-related findings should be interpreted cautiously, as further validation in established HCC models remains necessary.

## 1 Introduction

The present study focuses on zinc finger protein 473 (ZNF473), a factor implicated in multiple malignancies, with particular attention to its potential involvement in tumor progression and immune modulation. Cancer continues to represent a leading cause of morbidity and mortality worldwide, placing a sustained economic and societal burden on healthcare systems. Although established treatment modalities, including chemotherapy, radiotherapy, and targeted therapy, have improved clinical outcomes, durable benefit remains limited in many cases due to treatment resistance and toxicity. These limitations underscore the ongoing need to identify reliable biomarkers and actionable therapeutic targets (1).

Accumulating evidence suggests that ZNF473 may contribute to malignant progression; however, its biological roles and clinical significance have not yet been comprehensively defined. To avoid an arbitrary transition from a pan-cancer survey to a single-cancer model, the present study used a stepwise design. We first evaluated ZNF473 expression patterns, immune/molecular subtype associations, diagnostic performance, and survival relevance across cancers. HCC was then selected for deeper investigation because it showed convergent evidence across multiple analytical layers in this dataset, including significant upregulation, diagnostic performance, survival and Cox associations, clinicopathological relevance, and pathway-level links to phosphoinositide 3-kinase (PI3K)/AKT signaling. Recent studies in HCC have also emphasized the importance of molecular subtype stratification, prognostic modeling, and target-oriented therapeutic frameworks in refining risk assessment and guiding treatment selection, providing additional context for the present work (2–4).

The analytical strategy followed a stepwise workflow comprising pan-cancer screening of expression patterns and clinical relevance, evaluation of immune and molecular subtype associations, HCC-focused downstream bioinformatics analyses, and subsequent functional validation. Protein interactions involving ZNF473 were identified using the STRING database (5). The resulting protein–protein interaction (PPI) network was visualized in Cytoscape, followed by Gene Ontology (GO) and Kyoto Encyclopedia of Genes and Genomes (KEGG) enrichment analyses (6, 7).

ZNF473 expression across normal tissues and cancer types was derived from Genotype-Tissue Expression (GTEx)- and The Cancer Genome Atlas (TCGA)-based datasets. Diagnostic and prognostic performance were evaluated using receiver operating characteristic (ROC) curve analysis, Kaplan–Meier survival analysis, and Cox proportional hazards regression (8). To minimize arbitrary emphasis, tumor types were highlighted only when they demonstrated statistically significant expression differences and/or clinically meaningful diagnostic or prognostic associations. Among these, HCC was selected for in-depth analysis because it remained consistently significant across multiple analytical layers.

## 2 Method

### 2.1 GO and KEGG enrichment analysis of ZNF473

**Figure 1:**
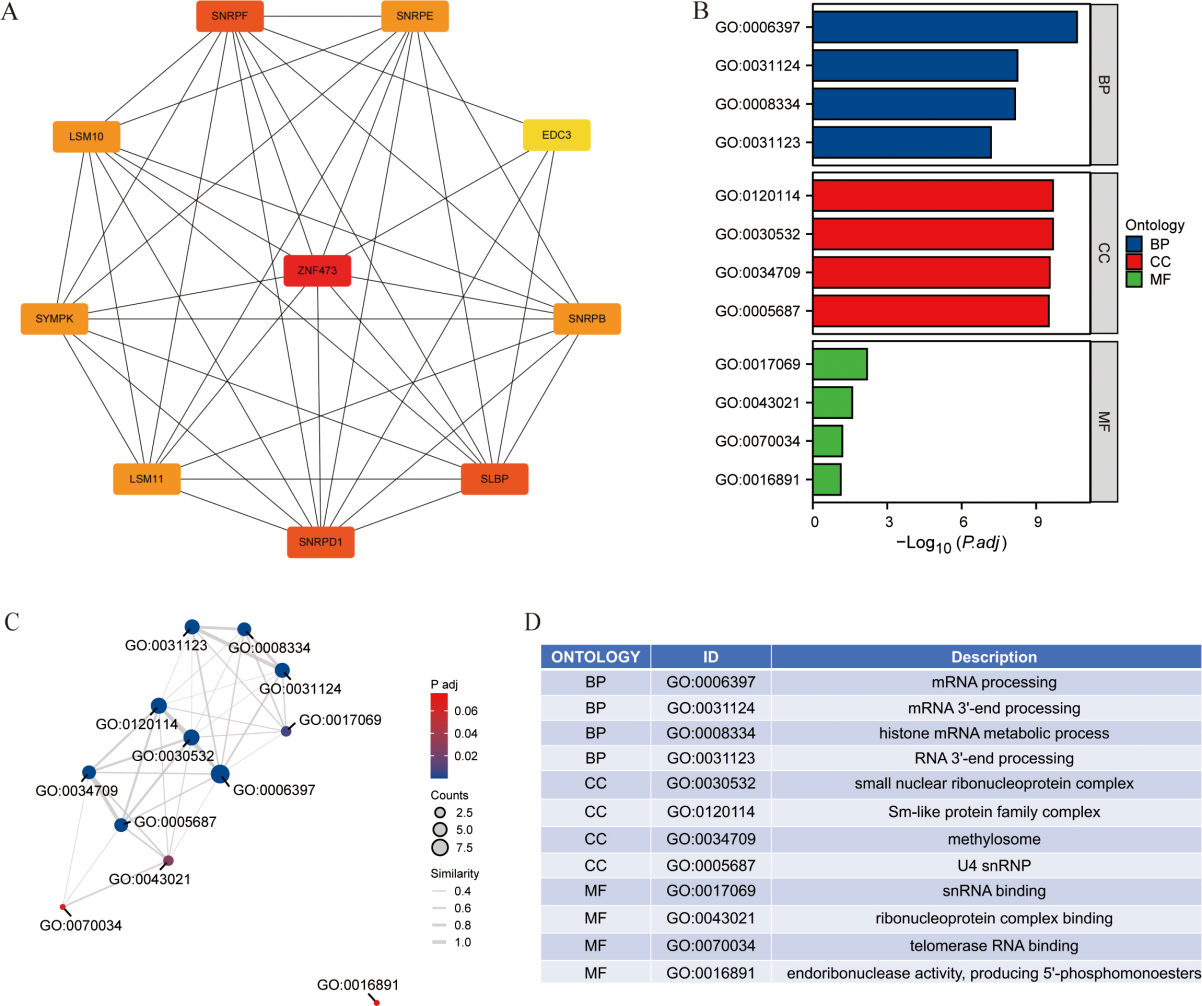
PPI, Protein–protein interaction network and GO, Gene Ontology analysis of ZNF473-interacting proteins. **(A)** PPI network. **(B)** GO enrichment analysis. **(C, D)** Network visualization of GO enrichment results.

Proteins interacting with ZNF473 were retrieved from the STRING database (https://string-db.org) by selecting interaction sources that included text mining, experimental evidence, and curated databases. The minimum required interaction score was set at 0.400 (medium confidence), and the maximum number of first-shell interactors was limited to 50 (9). The resulting interaction set was used as the predefined input for the PPI network construction and subsequent enrichment analyses presented in **Figure 1**.

The PPI network was visualized using Cytoscape (version 3.10.1). GO enrichment analysis of ZNF473-interacting proteins was performed using the clusterProfiler package (version 3.14.3), with graphical representation of enrichment results generated via the ggplot2 package (version 3.3.3) (10).

### 2.2 ZNF473 expression analysis

Expression data for cell lines and tissues were obtained from the Human Protein Atlas (HPA) database (https://www.proteinatlas.org) (11). RNA sequencing data were retrieved from TCGA and the GTEx databases via the UCSC XENA platform. Expression values, reported as transcripts per million (TPM), were uniformly processed using the Toil pipeline.

The pan-cancer analysis included all tumor types with available data across these resources. For figure presentation, emphasis was placed on tumor types demonstrating statistically significant differential expression and/or clinically meaningful diagnostic or prognostic relevance.

All statistical analyses were conducted using R software, and comparisons between groups were performed using the Wilcoxon rank-sum test. with P < 0.05 considered statistically significant.

### 2.3 ZNF473 expression across cancer subtypes

Associations between ZNF473 expression and both molecular and immune subtypes across cancers were evaluated using the Tumor–Immune System Interaction Database (TISIDB), an integrated resource that compiles tumor–immune interaction data from published studies and multiple high-throughput datasets (12, 13). These analyses were undertaken to assess whether ZNF473 expression varied significantly across predefined immune-related categories and to explore potential links between ZNF473 expression and the tumor immune contexture.

Given that TISIDB is an association-based platform, the findings from these analyses were interpreted as correlative rather than indicative of underlying mechanisms.

### 2.4 Diagnostic and prognostic evaluation of ZNF473 in pan-cancer

The diagnostic performance of ZNF473 across multiple cancer types was assessed using receiver operating characteristic (ROC) curve analysis (14, 15). Kaplan–Meier survival analysis and Cox regression were subsequently performed to identify tumor types with statistically significant prognostic associations.

The immune/subtype analyses were displayed because they served as exploratory association analyses across tumors with available subtype annotations. Subsequently, survival-focused presentation was restricted to tumor types that showed statistically significant associations in Kaplan–Meier and Cox analyses. This stepwise screening strategy was then used to justify selection of HCC for further validation.

### 2.5 ZNF473 expression and prognostic analysis across clinical subgroups in HCC

ZNF473 expression across patients with differing clinical characteristics in HCC was visualized using box plots and summarized in tabular form. RNA sequencing data and corresponding clinical information were obtained from TCGA, provided in level 3 HTSeq fragments per kilobase per million format. These data were subsequently converted to TPM and log2-transformed prior to analysis.

Comparisons between two groups were performed using the Wilcoxon rank-sum test, with statistical significance defined as *p* < 0.05 (ns, *p* ≥ 0.05; *, *p* < 0.05; **, *p* < 0.01; ***, *p* < 0.001). Associations between ZNF473 expression and survival outcomes, including overall survival (OS), disease-specific survival (DSS), and progression-free interval (PFI), were further assessed across clinical subgroups of patients with HCC (16, 17).

### 2.6 Immune infiltration and co-expression gene analysis of ZNF473 in HCC

Immune infiltration in relation to ZNF473 expression in HCC was evaluated using single-sample gene set enrichment analysis (ssGSEA), implemented through the GSVA package in R (version 3.6.3) (18, 19). This method was used to quantify enrichment scores for 24 immune cell types, as defined in prior studies. Spearman’s rank correlation analysis was then applied to assess associations between ZNF473 expression and immune cell enrichment scores.

In parallel, the top 50 genes co-expressed with ZNF473 in HCC, including both positively and negatively correlated genes, were identified. Gene co-expression patterns were visualized using heatmaps generated with the stats package. Pearson’s correlation analysis was further conducted to evaluate the relationships between ZNF473 expression and the expression levels of the top 10 co-expressed genes shown in the heatmap.

### 2.7 Differential gene expression analysis between high and low ZNF473 expression groups in HCC

Differentially expressed genes (DEGs) were identified between high and low ZNF473 expression groups in HCC, defined as the upper and lower 50% of expression levels, respectively, using the DESeq2 package (version 1.26.0). Volcano plots were generated with the ggplot2 package, applying thresholds of an absolute log2 fold change > 1.5 and *p* < 0.05 to define statistical significance.

GO and KEGG enrichment analyses of the identified DEGs were subsequently performed using the clusterProfiler package, with results visualized using ggplot2. A PPI network for these DEGs was constructed using the STRING database. Hub genes were then identified using the maximal clique centrality algorithm implemented in the CytoHubba plugin of Cytoscape (version 3.9.1).

### 2.8 Experimental validation *in vitro* and *in vivo*

Liver tumor–derived HepG2 cells and HCC–derived MHCC97H cells were obtained from the Cell Resources Center of the Shanghai Institute of Life Sciences. Cell proliferation was assessed using a Cell Counting Kit-8 (CCK-8) assay in accordance with the manufacturer’s instructions. Colony-forming capacity was evaluated using a colony formation assay. Cell migration was examined through scratch wound-healing and Transwell migration assays. Protein expression levels were analyzed by Western blotting, with antibodies against AKT and phosphorylated AKT (p-AKT) purchased from Abmart (Shanghai, China).

Given that HepG2 cells have been reported to exhibit hepatoblastoma-like features in certain classification frameworks, findings derived from this model were interpreted with caution and considered in combination with results obtained from MHCC97H cells.

Male BALB/c-nu/nu nude mice (4–6 weeks old, SPF grade; purchased from Charles River Laboratories) were maintained under controlled temperature and humidity conditions with a 12-h light/dark cycle and provided food and water ad libitum. All animal procedures were approved by the Animal Ethics Committee of the First Hospital of Shanxi Medical University.

For the subcutaneous xenograft experiment, HepG2 cells were transduced with lentiviral vectors to generate a control (Vector) group and a ZNF473-silenced group (si-ZNF473). Because HepG2 has recognized classification ambiguity, this xenograft assay was designed as supportive *in vivo* evidence and was not interpreted as definitive HCC-specific validation. Cells in the logarithmic growth phase were collected, washed with PBS, resuspended, and counted. The cell suspension was mixed with Matrigel (Corning) at a 1:1 (v/v) ratio to prepare an inoculum containing 5 × 10^6^ cells in a total volume of 100 μL. The prepared Vector or si-ZNF473 cells were then injected subcutaneously into the right dorsal flank of nude mice (n = 5 per group).

Mice were monitored regularly for general condition and tumor development, with tumor establishment assessed by palpation 1 week after injection. Tumor length (L) and width (W) were measured at designated time points, and tumor volume was calculated as *V* = (L × W²)/2. Mice were euthanized at 2 weeks post-inoculation or when tumor volume exceeded 2,000 mm³. Tumors were subsequently excised, photographed, and weighed.

## 3 Results

### 3.1 Pan-cancer functional context and expression characteristics of ZNF473

**Figure 1A** shows the PPI network of ZNF473-interacting proteins, highlighting links with multiple RNA processing-related factors. **Figure 1B** summarizes the major GO terms enriched among these interactors, whereas **Figures 1C, D** visualize the relationships among enriched terms and genes. Together, these panels indicate that ZNF473 is functionally associated with RNA metabolism and splicing-related processes, providing a biologically plausible framework for its involvement in cancer.

**Figure 2:**
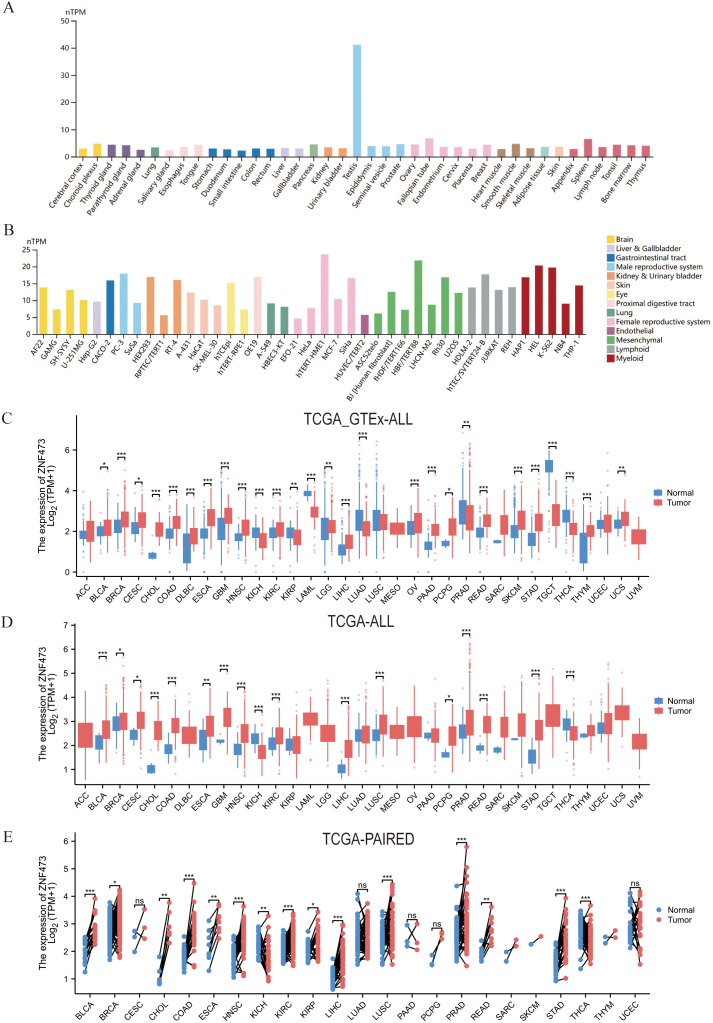
Expression patterns of ZNF473 across normal tissues and cancers. **(A)** ZNF473 expression in normal tissues. **(B)** ZNF473 expression in cell lines. **(C)** ZNF473 expression in TCGA tumors and normal tissues, with GTEx data used as controls. **(D)** ZNF473 expression in TCGA tumors and normal tissues. **(E)** ZNF473 expression in paired tumor and adjacent normal tissues. ns, not significant; *p < 0.05; **p < 0.01; ***p < 0.001.

**Figure 3:**
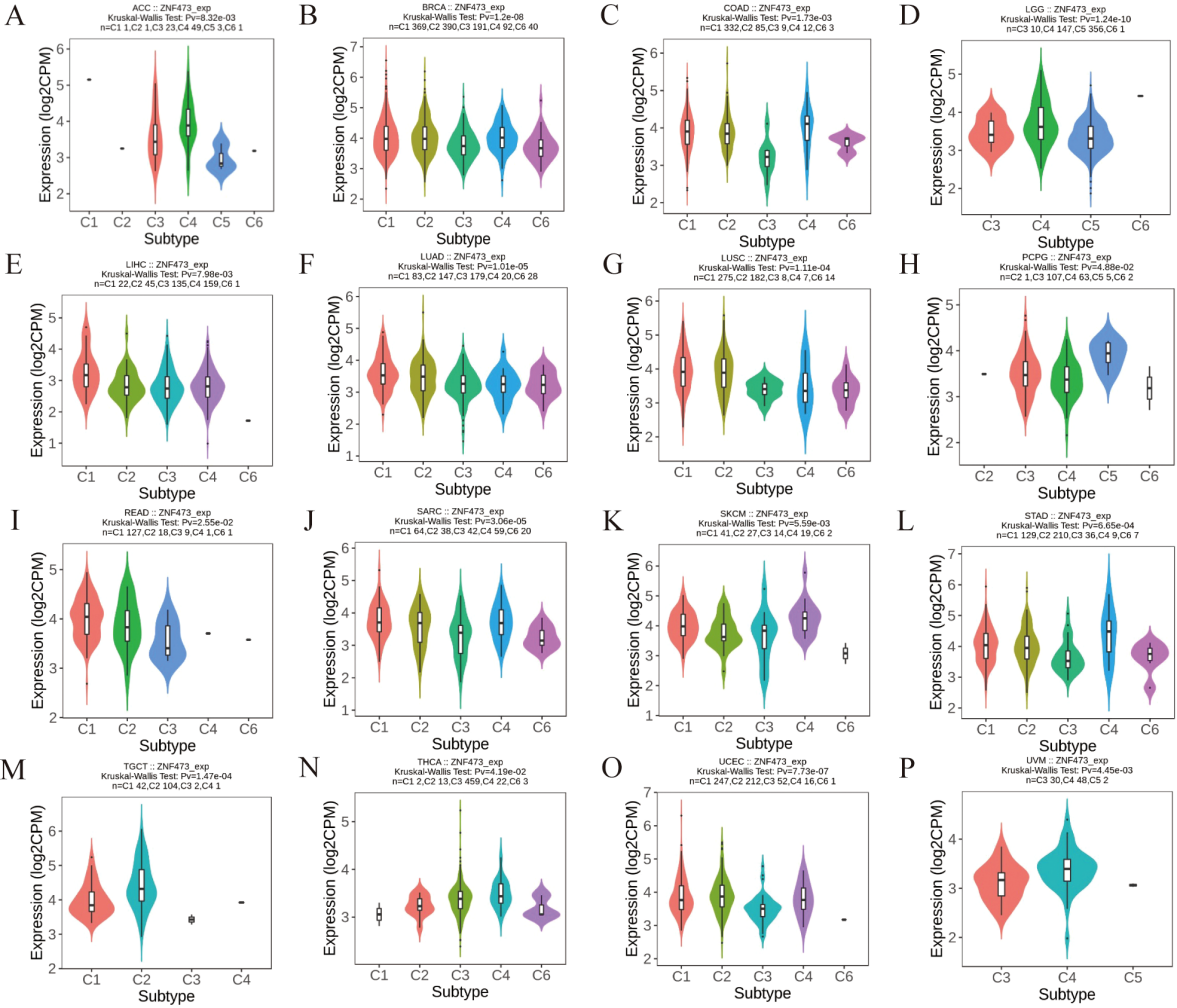
Associations between ZNF473 expression and immune subtypes across TCGA tumors. **(A)** ACC. **(B)** BRCA. **(C)** COAD. **(D)** LGG. **(E)** HCC. **(F)** LUAD. **(G)** LUSC. **(H)** PCPG. **(I)** READ. **(J)** SARC. **(K)** SKCM. **(L)** STAD. **(M)** TGCT. **(N)** THCA. **(O)** UCEC. **(P)** UVM. C1, wound healing; C2, IFN-γ dominant; C3, inflammatory; C4, lymphocyte-depleted; C5, immunologically quiet; C6, TGF-β dominant.

**Figure 2** presents expression data in multiple tumors. Specifically, **Figures 2A, B** show baseline ZNF473 expression across normal tissues and cell lines, **Figures 2C, D** compare tumor and normal tissues in the TCGA/GTEx-integrated and TCGA-only datasets, and **Figure 2E** summarizes paired tumor-adjacent comparisons. Across these expression panels, ZNF473 was significantly dysregulated in multiple tumor types, with notable upregulation in several malignancies, including HCC, and downregulation in kidney renal clear cell carcinoma (KIRC). **Figure 3** then shows that ZNF473 expression varied significantly across immune subtypes in multiple cancers, supporting an association with tumor immune contexture.

These association-based observations provided the rationale for subsequent HCC-focused analyses, particularly with respect to immune infiltration and related signaling pathways.

### 3.2 Diagnostic and prognostic screening identifies HCC as a priority cancer type

**Figure 4:**
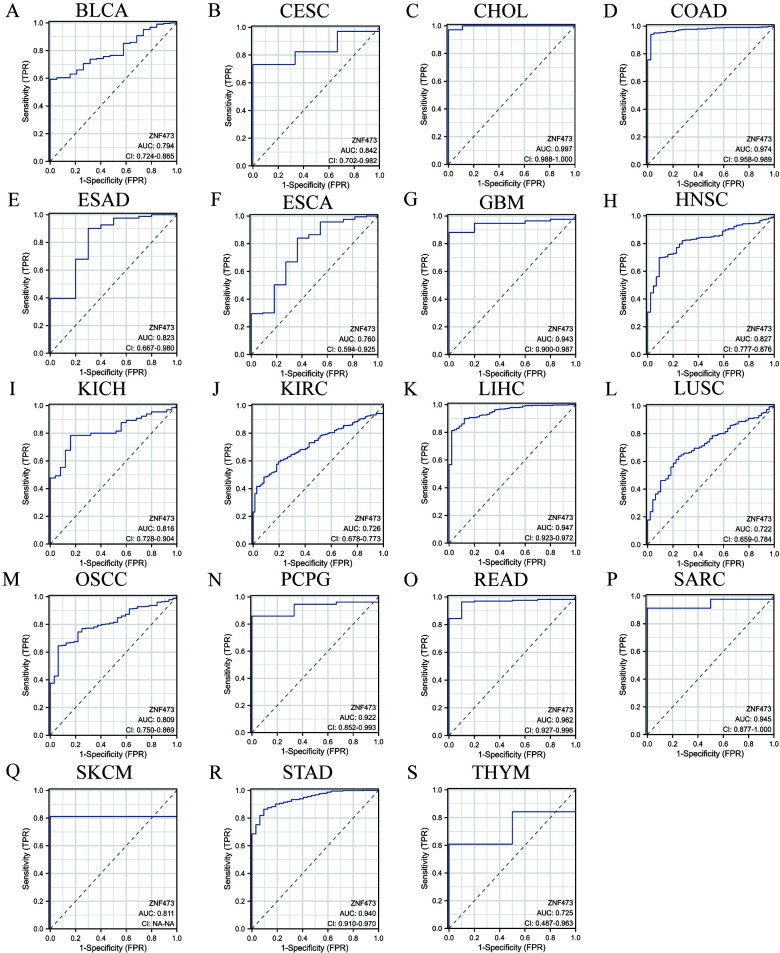
ROC curves for ZNF473 expression across cancers. **(A)** BLCA. **(B)** CESC. **(C)** CHOL. **(D)** COAD. **(E)** ESAD. **(F)** ESCA. **(G)** GBM. **(H)** HNSC. **(I)** KICH. **(J)** KIRC. **(K)** HCC. **(L)** LUCS. **(M)** OSCC. **(N)** PCPG. **(O)** READ. **(P)** SARC. **(Q)** SKCM. **(R)** STAD. **(S)** THYM.

**Figure 5:**
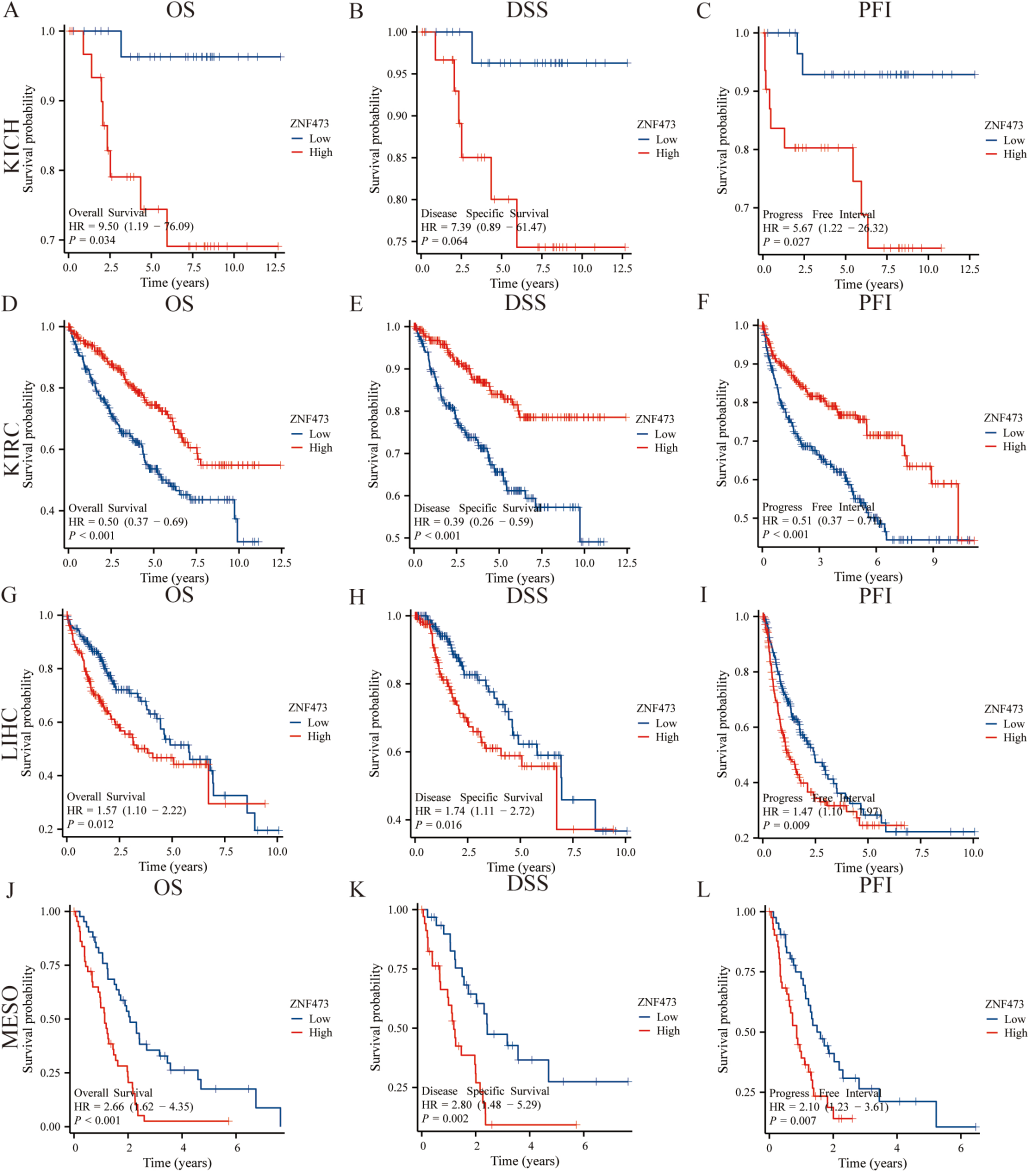
Associations between ZNF473 expression and survival outcomes (OS, DSS, and PFI) across cancers. **(A–C)** KICH. **(D–F)** KIRC. **(G–I)** HCC. **(J–L)** MESO.

ROC curve analysis demonstrated that ZNF473 achieved an area under the curve (AUC) > 0.7 in 19 cancer types, indicating moderate-to-high diagnostic performance across a broad pan-cancer context (**Figure 4**). Kaplan–Meier analyses of OS, DSS, and PFI identified significant associations in kidney chromophobe (KICH), kidney renal clear cell carcinoma (KIRC), HCC, and mesothelioma (MESO) (**Figure 5**).

Among these tumor types, HCC exhibited the most consistent pattern across analytical layers in the present study, including significant upregulation, diagnostic performance, survival associations, and subsequent clinicopathological and pathway-level relevance. On this basis, HCC was prioritized for further validation. Elevated ZNF473 expression in HCC was associated with poorer clinical outcomes, whereas an inverse association was observed in KIRC.

Cox proportional hazards regression analysis was subsequently performed to further assess the prognostic significance of ZNF473 in these malignancies. ZNF473 expression was associated with an increased risk of disease progression in HCC (**Table 1**), while a protective association was observed in KIRC (**Supplementary Table S1**).

**Table 1:** Univariate and multivariate cox regression analyses of ZNF473 in HCC.

Characteristics	Total (N)	Univariate analysis		Multivariate analysis	
		Hazard ratio (95% CI)	*P*-value	Hazard ratio (95% CI)	*P*-value
Age	373				
≤ 60	177	Reference			
> 60	196	1.205 (0.850 - 1.708)	0.295		
Sex	373				
Female	121	Reference	–	–	
Male	252	0.793 (0.557 - 1.130)	0.200		
Race	361				
Asian	159	Reference			
Black or African American	17	1.585 (0.675 - 3.725)	0.290		
White	185	1.323 (0.909 - 1.928)	0.144		
Pathologic Stage	349				
Stage I–II	259	Reference		Reference	
Stage III–IV	90	2.504 (1.727 - 3.631)	**< 0.001**	2.437 (1.678 - 3.538)	**< 0.001**
Histologic Grade	368				
G1–G2	233	Reference			
G3–G4	135	1.091 (0.761 - 1.564)	0.636		
ZNF473 Expression	373				
Low	186	Reference		Reference	
High	187	1.566 (1.105 - 2.221)	**0.012**	1.466 (1.009 - 2.130)	**0.045**

Bold values indicate statistically significant P-values (P < 0.05).

### 3.3 HCC-specific clinicopathological, immune, and pathway associations of ZNF473

**Figure 6:**
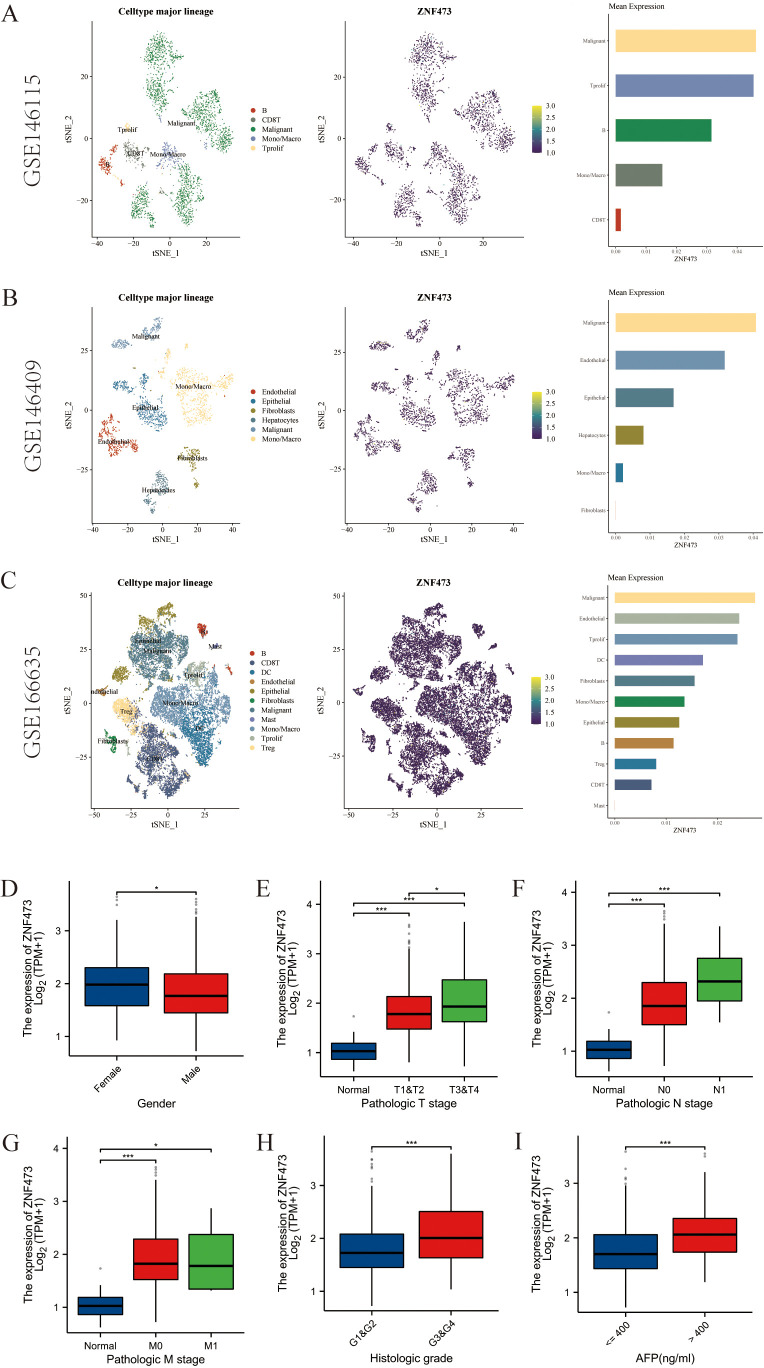
ZNF473 expression in single-cell datasets and clinicopathological subgroups of HCC. **(A–C)** Single-cell expression in HCC datasets: **(A)** GSE146115; **(B)** GSE146409; **(C)** GSE166635. **(D–I)** Expression across clinical subgroups: **(D)** sex; **(E)** T stage; **(F)** N stage; **(G)** M stage; **(H)** histologic grade; **(I)** AFP level. (*p* < 0.05; *p < 0.01; **p < 0.001).

**Figure 7:**
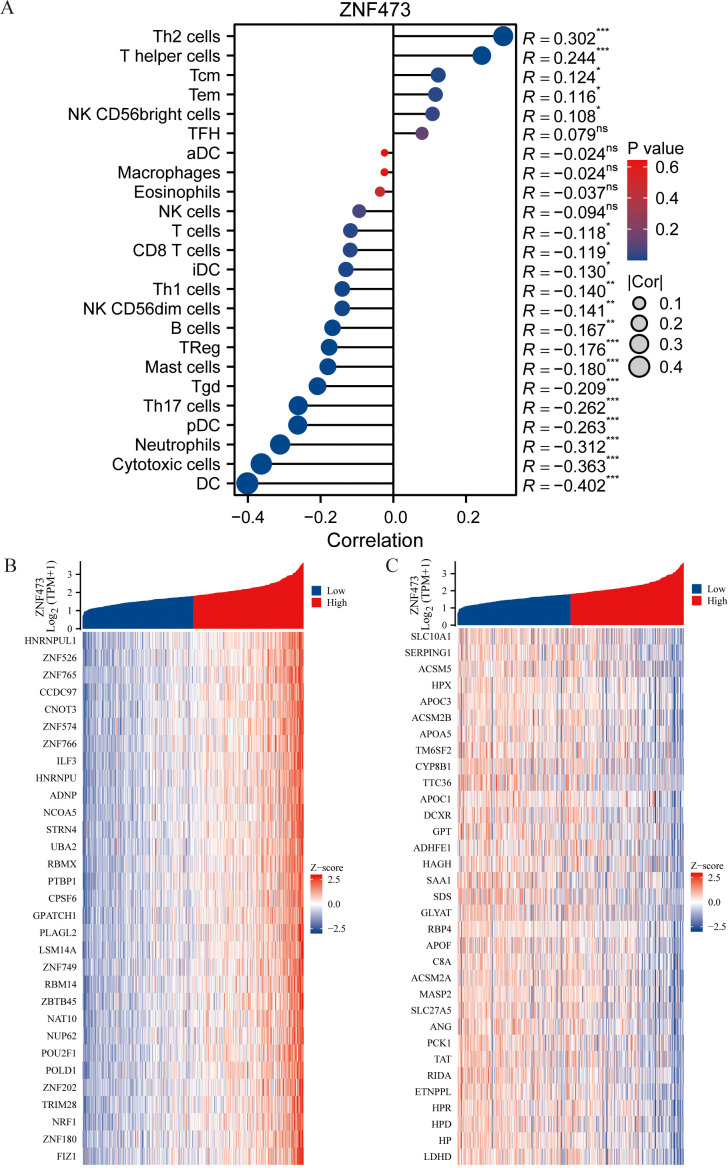
Immune infiltration and gene co-expression analysis of ZNF473 in HCC. **(A)** Forest plot showing correlations between ZNF473 expression and immune cell signatures. **(B)** Heatmap of positively correlated genes. **(C)** Heatmap of negatively correlated genes. ns, *p* ≥ 0.05; *p < 0.05; **p < 0.01; ***p < 0.001.

**Figures 6A-C** show that ZNF473 was detectable in three independent single-cell HCC datasets (GSE146115, GSE146409, and GSE166635). **Figures 6D-I** further show that higher ZNF473 expression in TCGA-HCC was associated with more adverse clinicopathological variables, particularly T stage, histologic grade, and AFP-related subgroups. **Figure 7A** demonstrates significant correlations between ZNF473 expression and multiple immune cell signatures, while **Figures 7B, C** display the top positively and negatively co-expressed genes in HCC.

**Figure 8:**
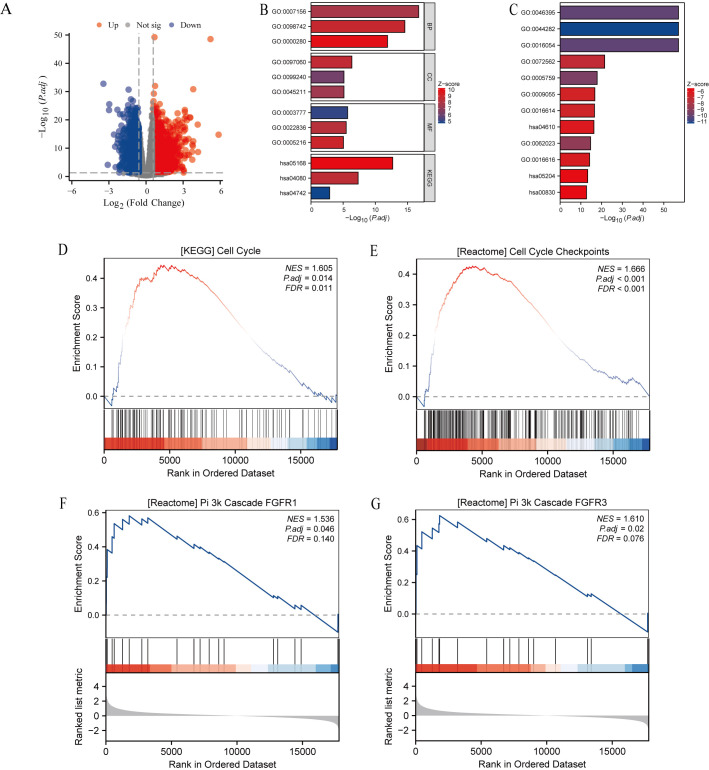
Differential expression and enrichment analysis between high- and low-ZNF473 expression groups in HCC. **(A)** Volcano plot of DEGs, differentially expressed genes (red, upregulated; blue, downregulated). **(B, C)** Network visualization of enrichment analyses. **(D–G)** GSEA, Gene set enrichment analysis of DEGs.

To further relate these observations to potential mechanisms, patients with HCC were stratified into high- and low-expression groups based on ZNF473 levels. **Figure 8A** shows the volcano plot of differentially expressed genes, **Figures 8B, C** visualize the enrichment/network relationships, and **Figures 8D–G** highlight PI3K-related pathways, including REACTOME_PI_3K_CASCADE_FGFR1 and REACTOME_PI_3K_CASCADE_FGFR3. These HCC-specific findings provided a basis for examining AKT pathway involvement in subsequent functional experiments.

These HCC-specific findings provided a basis for examining AKT pathway involvement in subsequent functional experiments.

### 3.4 Biological effects of ZNF473 in HCC

**Figure 9:**
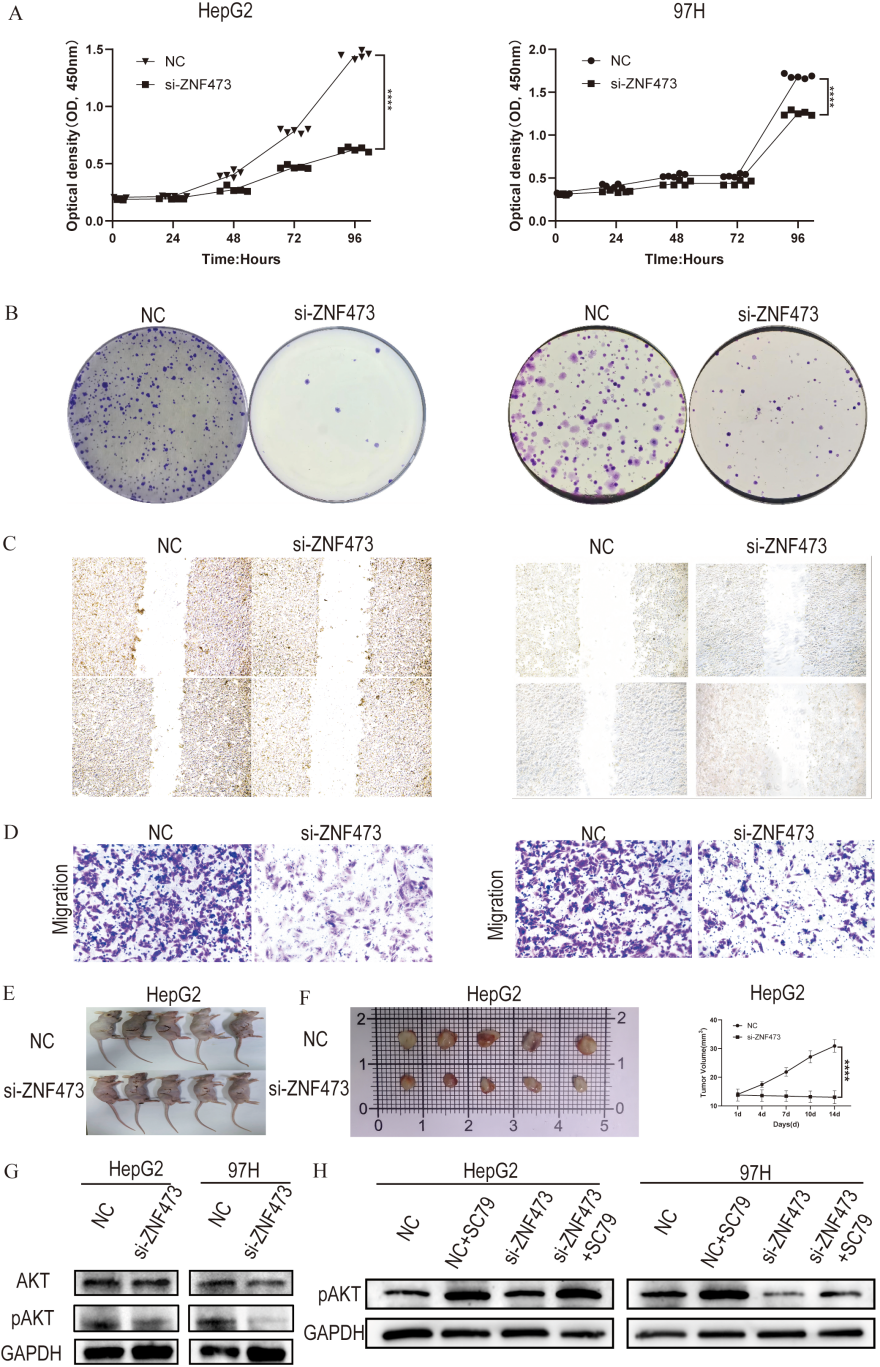
Functional effects of ZNF473 knockdown in liver tumor and HCC cell models. **(A)** CCK-8 assays in HepG2 and MHCC97H cells. **(B)** Colony formation assays in HepG2 and MHCC97H cells. **(C)** Wound-healing assays in HepG2 and MHCC97H cells. **(D)** Transwell migration assays in HepG2 and MHCC97H cells. **(E)** Representative nude mice in control and si-ZNF473 groups. **(F)** Excised xenograft tumors. **(G)** Changes in AKT and p-AKT, phosphorylated AKT levels following ZNF473 knockdown in HepG2 and MHCC97H cells. **(H)** Changes in AKT and p-AKT levels following ZNF473 knockdown combined with SC79 treatment in HepG2 and MHCC97H cells.

**Figures 9A, B** show that ZNF473 knockdown reduced proliferative capacity in HepG2 and MHCC97H cells in the CCK-8 and colony formation assays. **Figures 9C, D** show impaired migration in the wound-healing and Transwell assays. **Figures 9E, F** present the HepG2-derived subcutaneous xenograft results as supportive *in vivo* evidence, while **Figures 9G, H** show that ZNF473 knockdown reduced p-AKT and that SC79 partly restored AKT activation. Because the xenograft model was established with HepG2 cells, these *in vivo* data were interpreted cautiously and were not used as the sole basis for the HCC-specific conclusion.

A decrease in p-AKT levels following ZNF473 knockdown, together with partial recovery after SC79 treatment, supported the involvement of the AKT signaling pathway in these effects.

Given the model-specific considerations associated with HepG2 cells, the HepG2-derived results should be regarded as supportive liver tumor evidence, whereas the HCC-related interpretation in this study is primarily supported by the combined MHCC97H experiments and TCGA-HCC analyses. Further validation in additional authenticated HCC models remains warranted.

## 4 Discussion

The present study employed an integrated workflow combining bioinformatics analyses with experimental validation to assess the biological and clinical relevance of ZNF473 across cancers, with particular focus on hepatocellular carcinoma (HCC). The pan-cancer component was used to determine whether ZNF473 showed broad expression and clinical relevance, whereas the HCC-focused component was undertaken only after HCC emerged as the tumor type with the most internally consistent signal across this dataset. This selection was supported by multiple lines of evidence, including differential expression, diagnostic performance, survival associations, clinicopathological correlations, immune-related analyses, and pathway-level enrichment.

Specifically, HCC combined significant upregulation, acceptable diagnostic discrimination, survival and Cox relevance, clinicopathological associations, immune correlations, and PI3K/AKT-related enrichment, which together justified the downstream experimental validation (20, 21). Such an approach helps maintain continuity between the initial pan-cancer screening and the subsequent HCC-specific validation experiments, reducing the risk of arbitrary selection and strengthening the overall coherence of the study design.

### 4.1 ZNF473 as a biologically and clinically relevant target in cancer

Investigation of ZNF473 is of particular relevance in oncology, where dysregulated gene expression and altered protein interactions play a central role in tumor progression and clinical outcomes. As a zinc finger protein, ZNF473 has been associated with key biological processes, including RNA processing and the regulation of gene expression—both of which are frequently disrupted in malignant contexts (22). Accordingly, examining the role of ZNF473 in tumor biology is warranted, as its expression patterns and functional associations suggest potential utility as a diagnostic and prognostic biomarker across multiple malignancies (23, 24). Further clarification of the molecular pathways linked to ZNF473 may also inform the development of targeted therapeutic strategies aimed at improving patient outcomes.

In the present study, ZNF473 expression patterns, PPI networks, and prognostic relevance were systematically evaluated across cancer types. Functional enrichment analyses highlighted biological processes and pathways potentially associated with ZNF473, while diagnostic and prognostic assessments, including ROC curve analysis and Kaplan–Meier survival analysis, provided additional context for its clinical relevance. The findings indicate that ZNF473 is associated with multiple aspects of tumor biology, including RNA processing and immune-related processes, suggesting potential relevance for therapeutic development.

Notably, ZNF473 showed consistent associations with RNA processing and splicing-related pathways. Enrichment analyses further indicated involvement in messenger RNA processing and histone messenger RNA metabolism, processes that are important for maintaining genomic stability and accurate gene expression. Aberrant RNA splicing is widely recognized as a feature of cancer, and these observations raise the possibility that ZNF473 may contribute to tumorigenesis through modulation of RNA splicing–related mechanisms.

In addition, expression profiling revealed marked upregulation of ZNF473 in several malignancies, including HCC, as well as downregulation in kidney renal clear cell carcinoma. These tumor-specific expression patterns may have potential relevance for diagnostic and prognostic assessment (25).

### 4.2 Clinical relevance of ZNF473 in cancer

Diagnostic evaluation indicated that ZNF473 exhibited moderate-to-high accuracy across multiple cancer types, with AUC values exceeding 0.7 in 19 tumors, supporting its potential relevance as a biomarker. The HCC-focused findings are also in line with recent studies highlighting the role of molecular stratification and prognostic modeling in HCC, including work on programmed cell death–related risk architectures and BRD4-associated therapeutic vulnerability (3, 4).

Beyond diagnostic performance, ZNF473 expression showed associations with clinical outcomes, including OS, DSS, and PFI in selected cancers. Among these, HCC demonstrated a relatively consistent pattern across both clinical and molecular analyses. Cox proportional hazards regression further indicated prognostic relevance, with reduced ZNF473 expression associated with poorer survival outcomes, whereas the direction of association was reversed in KIRC (26).

These observations are of particular interest in HCC, where robust biomarkers for prognosis and treatment guidance remain limited. Taken together, the findings point to a potential clinical role for ZNF473 in cancer biology and support continued investigation into its underlying mechanisms and possible therapeutic relevance (27, 28).

### 4.3 ZNF473 and PI3K/AKT signaling in HCC

The findings indicate that ZNF473 is associated with regulation of the AKT signaling pathway, which plays a central role in cell growth, survival, and metabolism (29–31). HCC-specific enrichment analyses pointed to PI3K-related signaling, providing a link between the bioinformatics observations and the reduction in p-AKT following ZNF473 knockdown (32). While these results support a role for ZNF473 in modulating AKT activation, they do not establish a direct mechanistic relationship (33, 34).

Restoration of p-AKT levels after treatment with SC79, an AKT activator, further suggests that the effects of ZNF473 are mediated, at least in part, through the AKT signaling cascade (35). At the same time, the precise molecular basis of this regulation remains unclear. It is not yet evident whether ZNF473 directly interacts with AKT or acts through upstream regulators such as PI3K, and this distinction will require further investigation (36).

These observations contribute to a broader understanding of how AKT phosphorylation may be influenced in HCC and highlight the potential relevance of this pathway in ZNF473-associated effects. Given the established role of AKT signaling in tumor progression and metastasis, clarification of how ZNF473 interfaces with this pathway may help identify additional points for therapeutic intervention.

The impact of ZNF473-associated regulation of the AKT pathway appears to extend beyond effects on cell proliferation and migration. Given the well-established role of AKT signaling in tumor progression and metastasis, further clarification of how ZNF473 influences this pathway may help identify additional therapeutic targets. Future studies should aim to define the broader signaling networks involving ZNF473 and AKT, as well as to evaluate whether combined targeting of ZNF473 and AKT pathway components offers therapeutic benefit in cancer.

Several limitations should be considered. Although subcutaneous xenograft experiments were conducted, orthotopic and metastasis models were not included; accordingly, the *in vivo* findings may not fully reflect the complexity of HCC progression. In addition, HepG2—used as one of the *in vitro* and *in vivo* models—has recognized classification ambiguity and may not adequately represent canonical HCC biology. The *in vivo* model was HepG2-derived and therefore should not be overinterpreted as definitive HCC evidence. The functional findings therefore warrant validation in additional authenticated HCC cell lines, such as Huh7 and Hep3B, ideally with short tandem repeat profiling and inclusion of normal hepatocyte controls. The sample size for experimental validation was limited, and independent external clinical validation was not performed. These limitations should be addressed in future work.

## 5 Conclusion

The present findings indicate that ZNF473 is associated with malignant phenotypes and activation of the AKT signaling pathway in liver tumor and HCC-related models. In particular, the combined bioinformatics analyses and MHCC97H experiments support ZNF473 as a biomarker candidate and potential therapeutic target in HCC, whereas the HepG2-derived findings should be interpreted as supportive rather than definitive. Reduced ZNF473 expression was associated with decreased proliferative capacity and migratory ability, accompanied by lower levels of p-AKT.

These observations support a role for ZNF473 in tumor biology and point to its possible relevance as a therapeutic target in cancer. Further studies are needed to clarify the underlying molecular mechanisms and to evaluate the clinical potential of targeting ZNF473 in oncological settings.

## Data Availability

The original contributions presented in the study are included in the article/**Supplementary Material**. Further inquiries can be directed to the corresponding authors.

## Glossary

HCC: hepatocellular carcinoma
OS: overall survival
DSS: disease-specific survival
PFI: progression-free interval
Pakt: phosphorylated AKT
ssGSEA: single-sample gene set enrichment analysis
ROC: receiver operating characteristic
DEGs: differentially expressed genes
GO: Gene Ontology
KEGG: Kyoto Encyclopedia of Genes and Genomes
snRNP: small nuclear ribonucleoprotein
AFP: alpha-fetoprotein.
