# Supplementary material for: ZNF473 as a biomarker and potential therapeutic target in cancer: integrated bioinformatics and experimental evidence with a focus on hepatocellular carcinoma
Source: Front Oncol. 2026 May 15;16:1810579. doi: 10.3389/fonc.2026.1810579 (PMC13218875; doi:10.3389/fonc.2026.1810579)
Supplement: Supplementary file 1 [file Table1.docx]

**Table S1 Univariate analysis and multivariate analysis of ZNF473 in KIRC**

| **Characteristics** | **Total(N)** | **Univariate analysis** | | **Multivariate analysis** | |
| --- | --- | --- | --- | --- | --- |
|  |  | **Hazard ratio**  **(95% CI)** | **P value** | **Hazard ratio**  **(95% CI)** | **P value** |
| Age | 541 |  |  |  |  |
| <= 60 | 269 | Reference |  | Reference |  |
| > 60 | 272 | 1.791 (1.319 - 2.432) | < 0.001 | 1.557 (1.144 - 2.119) | 0.005 |
| Gender | 541 |  |  |  |  |
| Female | 187 | Reference |  |  |  |
| Male | 354 | 0.924 (0.679 - 1.257) | 0.613 |  |  |
| Race | 534 |  |  |  |  |
| Asian | 8 | Reference |  |  |  |
| Black or African American | 57 | 1.578 (0.203 - 12.236) | 0.663 |  |  |
| White | 469 | 1.857 (0.259 - 13.292) | 0.538 |  |  |
| Pathologic stage | 538 |  |  |  |  |
| Stage I&Stage II | 332 | Reference |  | Reference |  |
| Stage III&Stage IV | 206 | 3.910 (2.852 - 5.360) | < 0.001 | 2.976 (2.131 - 4.155) | < 0.001 |
| Histologic grade | 533 |  |  |  |  |
| G1&G2 | 250 | Reference |  | Reference |  |
| G3&G4 | 283 | 2.665 (1.898 - 3.743) | < 0.001 | 1.619 (1.126 - 2.327) | 0.009 |
| ZNF473 | 541 |  |  |  |  |
| Low | 270 | Reference |  | Reference |  |
| High | 271 | 0.504 (0.371 - 0.686) | < 0.001 | 0.624 (0.455 - 0.857) | 0.004 |
